# Observations on the Scarlatina Anginosa

**Published:** 1806-06

**Authors:** Jonathan Binns

**Affiliations:** Physician at Lancaster, and Member of the Royal College of Physicians, London


### Medical and Phyfical Journal.

VOL. XV.] JUNE, 1806. [NO. 88.

*Printed for R. PHILLIPS, by W. Thorne, Red Lion Court, Fleet Street, London.*

### OBSERVATIONS *on the* SCARLATINA ANGINOSA; *by* JONATHAN BINNS, M. D. *Physician at Lancaster, and Member of the Royal College of Physicians, London.*

In a Letter addressed to Dr. WILLAN.

Dear Doctor,

IN thy Treatise on Scarlatina Anginosa, it appears as if I am the only person who supposes he has seen the disease occur twice in the same patient. I have heard that some other practitioners have observed it, but I shall quote a published authority or two, which, in thy extensive reading on the subject, thou hast probably overlooked. In the Med. and Phys. Journal, vol. xiii. p. 213, Dr. Hall, Clement's Inn, remarks, that "it has been supposed, that specific contagion can affect individuals only once during life; although the doctrine may hold true, in general, with regard to measles, small-pox, &c. it will not apply to other contagious diseases, as typhus, *scarlatina*, &c. to which, after some time, the same susceptibility returns as at first." And the late Dr. Heberden, treating on this subject in his Commentaries, Engl. edit. p. 28, has these words: "According to my experience, some children have, beyond all doubt, been afflicted a second time with this disease:" yet adds, "but it is evident this happens very seldom."

As the latter gentleman made notes during attendance on his patients, and frequently revised these notes, it is to be presumed he was correct. I could wish these testimonies to be brought forward, that I may not stand alone; and, if I have erred, it may appear that it was in the most respectable company: also, if there still remain a doubt, that the Public may not rest in security till the Faculty shall find, by further observations, that the general opinion is confirmed.

In the course of our correspondence I informed thee, that I could not say that my patients, who, I supposed, had had this disease oftener than once, had exactly the same symptoms on the repetition; I rather think they had not the rash on the surface, but they had apparently the kind of sore throat peculiar to this disease, with a considerable fever. That the rash is not an essential part of the disease I conclude from thy work; as, in pp. 274 and 281, it is acknowledged, that "the scarlet ulcerating sore throat, without the efflorescence on the skin, is a variety of this disease, and is capable of communicating, by infection, all the varieties of the disease." I suppose the following was a case of this kind, which lately occurred here during the time that the scarlatina anginosa was epidemic. A boy, about eleven years of age, was seized with an ulcerated sore throat, with increased redness of the fauces and of the papillæ of the tongue; he had a great degree of fever, an intense heat, and considerable redness of the skin about the neck and breast, but no rash. It was about the third day after the attack that I first saw him; he was immediately subjected to the affusion of cold water over the whole surface (perhaps this might tend to prevent the rash). This operation was repeated three times in ten hours, which had a good effect in moderating the heat, which never rose to the same height during the disease, notwithstanding the liberal use of bark, wine, and aromatic powder.

I do not conceive from the absence of the rash on the surface in this case, that it was not the same disease as the reigning epidemic; and I have been induced to suppose, when similar symptoms have occurred in persons who previously had undergone scarlatina anginosa, that they were of the same nature. It is not improbable that my cases were such as are mentioned in the third vol. of the Edinburgh Medical Essays, quoted by thee, p. 312, "that such patients as had undergone the scarlet fever at any time in their lives before, took at this time the fever and angina, without the scarlet eruption." But it does not appear by that Essay, whether any of *these* "were suffocated by the angina," or it was only such as had the scarlet eruption. We may however, I think, safely conclude, that both took the disease in the same way, probably by infection: if so, may we not also conclude, that the contagion of scarlatina anginosa is capable of communicating to them that have previously had it, a fever and angina? And should the secondary infection (if such be granted) never prove mortal, mortal, it surely is better, especially for the youth, to avoid it as much as well may be, not only for the sake of them who may be the immediate objects of it, but for fear it also may be contagious, and communicate the fever and angina to others, as well as the eruption. I have been so little accustomed to consider the rash as of material importance, except in characterizing the disease when other symptoms might render it doubtful, that it is probable my expression, inserted in page 283, "scarlet fever and sore throat," should rather have been, "the sort of fever and sore throat that usually attend scarlatina anginosa."

If my observations upon this subject should have no weight, I hope the authorities I have quoted, namely, Dr. Harrison, Dr. Heberden, and the paper in the Ed. Med. Essays, will have so much influence as to induce thee to caution the public against an implicit reliance on the doctrine pretty generally inculcated, that there is no risk of infection to them who have had the disease from an indiscriminate communication with persons laboring under the disease, or their attendants; and to request the Faculty to continue their observations, in order, by still more extended negative proof, to have the matter more certainly decided, that there is no danger of a second infection. If this shall prove to be the case, it will be gratifying to me, as I consider this disease as one of the most dreadful that afflicts this country.

From thy request to insert my communications in thy work, and from other parts of thy letters, I conceived that my practice in this disease had met thy approbation; but there is reason to think otherwise from thy expression in p. 366. "Whatever opinion may be formed respecting the above mode of practice during the first days of the fever, it is generally admitted that, at the decline of the efflorescence, if the fever also declines and is not succeeded by a cough, Peruvian bark, mineral acids, wine, and nutritious diet, obviate the debility and oppressive languor which remain after the disease." And in a Note on the same page, there is a quotation from Dr. Sims, saying, that "as soon as the pulse on the sixth day began to fall to the natural standard, if the cordial medicines and regimen were persisted in, or increased, with a view to keep up the sinking pulse, many vexatious or even dangerous consequences ensued.

When thus we find one of these eminent authors and physicians in the metropolis discountenances my mode of treating the disease on the first days, and the other when it it is on the decline, there seems just ground to suppose, that their readers will be induced to reject what is recommended by an obscure individual in the country. I am aware that several others have written against this plan; on the other hand, I think a few have approved of it: but I do not find that I have made any extracts from them, probably owing to my having been long convinced of the utility of the practice, and not expecting that I should have occasion to defend it; and I may mention, that I have had the satisfaction to receive, on this occasion, the congratulations of an eminent physician in extensive business, who had perused thy work. But as Doctors differ, and as the scarlet fever differs in all degrees, from a flea-bite to the plague,* it is difficult to determine, from the histories given, what practice is most successful, unless we could know the degrees of virulence. For example, the children at Ackworth, in 1803, had in general the disease much more severely than those who had it there about eight years before, very few of whom were in danger, and none died of the disease; so that, in some cases, as Dr. Fothergill observed, the disease is of so mild a nature that persons recover from it under unskilful management. I shall now endeavour to shew, by analogy, the reasonableness of my mode of practice in this disease, in order, if may be, to convince thee that I act on sound principles.

In this disease there are generally considerable *heat and inflammation on the surface;* † but it is also attended with *great debility,* and there is frequently a great tendency to *putrefaction and mortification.* Against all these symptoms the Peruvian bark appears to me a powerful medicine.—That accurate observer, the late John Hunter, in treating of mortification proceeding by inflammation, says, "the bark is the only medicine that can be relied on, as it *increases the powers, and lessens the action.*" And Murray, in the Materia Medica, speaking of bitters, observes, that "the bark *strengthens most, without increasing heat.*"

It is allowed by various Authors, that scarlatina anginosa bears much resemblance to erysipelas. When I was at Edinburgh, about thirty-five years ago, it was the practice to treat the latter disease as a purely inflammatory complaint, by bleeding and purging; I suppose it is still the

---

* Darwin's Zoonomia.

† Dr. Withering remarks on this disease, that the small vessels accumulate the blood there more in consequence of *their own action,* than from the pulse of the heart.

the case: and I am told that the other is now treated there by purgatives. But it is probable that in the north, diseases bear evacuations better than in the south; for on my visiting London, soon after leaving Edinburgh, I was informed by the late Doctor Fothergill, that he frequently found the antiphlogistic plan could not be borne there by patients in erysipelas, on account of its tendency to mortification; and that he generally experienced the best effects from bark and wine. But thy objection seems to lie chiefly against the use of these remedies during the first days of scarlatina anginosa, before the efflorescence and fever decline: but, I trust, the prejudice against giving the bark with a febrile pulse is on the decline. Dr. Wells, in the Transactions of a Society for the Improvement of Medical and Chirurgical Knowledge in London, informs us, that "no indirect arguments have been found necessary to persuade those to relinquish the practice of attempting to cure erysipelas by large evacuations, in London at least, who have witnessed the effects of the Peruvian bark, when *given early* in this disease *in large and frequent doses.* In a large woman, of a florid complexion, and constitution vigorous from nature, and unbroken by intemperance or disease, whom he saw a few hours after her face began to swell, he immediately prescribed for her the Peruvian bark, though *her pulse was frequent, her skin hot,* and a dry brown streak divided longitudinally the upper surface of her tongue; she took *four ounces of that medicine* in substance during the *first three days of her illness.* "This quantity," he remarks, is indeed not nearly so great as is sometimes exhibited in erysipelas by Dr. George Fordyce, who for upwards of twenty years has been accustomed to give, at St. Thomas's Hospital, a drachm of the Peruvian bark in powder every hour in dangerous states of the disease: but as this medicine, in the above case, was given almost from the commencement of her illness, less was probably required for her cure than would have been if her disease had made greater progress before it was exhibited." Even in Holland, where, thirty-four years ago, very few physicians, except Prof Gaubius, ventured to give the bark in intermittents, we since find, by Memoirs of Haarlem Society, that Dr. Verryst, a Dutch physician, gave it in malignant fevers to the quantity of *nine or ten ounces* within *three of the first days.* And Dr. Lafuente, Physician to the Spanish forces, has lately adopted the practice of "giving at least *six or eight ounces of bark* in the *first forty-eight hours* of the yellow fever; saying, it is

 absolutely absolutely necessary to take the bark as soon as the symptoms of the fever make their appearance; the practice recommended by Dr. L. is not altogether so new as he seems inclined to suppose, for it is well known that British practitioners, both at home and abroad, have long been in the habit of prescribing *cinchona* in various malignant fevers, from the very commencement of the disease, and in as large doses as the stomach can bear, without any regard whatever to the state of pyrexia.* The late Dr Heberden, in his Commentaries, expresses himself thus; "The Peruvian bark in fevers has been much dreaded except in a perfect intermission; but the free use of it, notwithstanding the height of the fever in mortification, &c. has taught us that this dread is groundless. It has been tried in high continual fevers, in which I am not so sure of its being useful, as I am of its being innocent, whether in decoction or powder;" and p. 185, "The only harm that I believe would follow from taking the bark, even in the middle of a fit (of the ague) is, that it might occasion a sickness, &c. Dr. Tavares, Archiatros of Portugal, remarks, "that the best authors advise, in the worst species of intermittents, even attended with inflammatory symptoms, to give the bark rather too early than omit it too long; and adds, that it is not possible to cure the worst species of intermittents unless a large quantity is given as well in the interval of remission or intermission, as *during the paroxysm itself.*† The same physician, as well as some others he quotes, gives the bark in the inflammatory state of the gout.‡ And a very respectable physician of our own country, following Dr. Morton, Sir E. Hulse, and Dr. Fothergill, gives the bark after an emetic in the *beginning* as well as in the further progress of one of the most highly inflammatory diseases, viz. the acute rheumatism.§ These authorities, I trust, are sufficient to shew the safety of giving the bark in a highly febrile state.

With respect to the exhibition of wine in scarlatina anginosa, I began to use it liberally many years ago, on being requested to visit the child of a person, who, in a very short space of time, had lost four out of six children, in this complaint, at a time in which it was epidemic

* Med. and Phys. Journal, Vol. 14, pp. 534 and 5.

† Med. and Phys. Journal, Vol 12, p. 153.

‡ Ibid

§ Haygarth's Clin. Hist. of Diseases.

demic in Liverpool; and this child also was considered as past recovery by the apothecary, who, on that account, had declined his visits. It was about the fourth day of the disease, and I do not recollect ever seeing a patient recover from a more dreadful state. I considered it as a hopeless case, but from the distress of the parents, I resolved to make an effort, and to give as much bark and wine as could be got down. Finding them agree with the little patient, who made no resistance to the means which were attempted for his relief, but appearing to revive, I increased the wine to a much greater quantity than I had before given to so young a child, who, as I recollect, was between three and four years of age; I think he took not less than a pint of red Port per day for about six successive days.

As these remedies, with frequent gargling, answered in this case beyond expectation, on future occasions I gave them more early, on a supposition that if the wine did not increase the fever and heat, it would, with the bark, have a tendency to support the strength and resist putrescence, circumstances which I consider of great importance in this disease; and in this, I trust, I was not deceived: but if we wait till the fever and efflorescence decline, I fear it will often be too late to attempt any remedies.

I find my remarks have extended much beyond the intended limits, but before I conclude I wish further to observe, that as various other stimulants appear to have been liberally given, and with advantage, in this depressing disease, I conceive, from both analogy and experience, that wine may, in most cases, be plentifully given, not only with safety but advantage, and conjoined with the bark, and nutritious diet when it can be taken, they are, in my opinion, likely to afford a more permanent support to the system than volatile alkali, or any other medicine with which I am acquainted.

As this kind of discussion seems rather unsuitable to be introduced into thy work on cutaneous diseases, I take the liberty to suggest whether it might be adviseable to request its admission into the Medical and Physical Journal; by which other practitioners may be incited to give their experience and observations on these important topics, and we at length be able to determine, which is the most efficacious mode of treating this virulent disease.

I am, &c.

JONATHAN BINNS.

*Lancaster, 22d of 3d Month,* 1806.

